# Navigating Masculinities: The Role of Internalised Oppression and Domination in Gendered Violence Amidst Anti-Gender Politics

**DOI:** 10.1177/10608265261422356

**Published:** 2026-02-02

**Authors:** Erman Örsan Yetiş

**Affiliations:** 1 School of Sociological Studies, Politics and International Relations, 7315 The University of Sheffield, Sheffield, UK

**Keywords:** gendered violence, symbolic violence, continuum thinking, conducive context, feminist social justice

## Abstract

This paper explores gendered violence through intricate dynamics of internalised oppression and domination within contexts where anti-genderism has arisen and subsequently exacerbated it. Turkey exemplifies the operation of anti-genderism linked to a majoritarian-authoritarian-securitarian top-down political agenda. Growing discontent against increasing social violence linked to this agenda signals potential for men’s involvement in feminist social justice endeavours against anti-gender politics. However, men may find it challenging to embrace a feminist perspective due to their contentious positionalities regarding power dynamics involving internalised oppression and domination. Drawing on continuum thinking about gendered power relations, the paper reveals complex barriers that prevent men from cultivating alternative, empowering political-ethical imaginations. For men to engage in feminist social justice struggles against anti-genderism, they initially require reckoning with these power dynamics.

Today, anti-gender movements present a global challenge, primarily asserting that the invention of “gender” as an ideology is eradicating the natural and inevitable differences between men and women in general. As a term coined and mobilised by anti-gender politics and movements to frame feminist, queer, and gender equality agendas as dangerous or imposed ideology, “gender ideology” is not a neutral academic concept but a polemical tool of opposition. While this opposition to the so-called gender ideology contains contradictions from the very beginning, the concept of “gender” as a category of analysis (Scott, 2010) and its political use are perceived as dangerous to the extent that it exposes and criticises the existing gender regimes and the unequal power relations established around them (Butler, 2024). In this regard, presenting “gender ideology” as a threat can be seen as asserting ontological control by dictating how gender is defined, interpreted, and institutionalised (Corredor, 2019). Anti-gender politics pose a serious threat to the rights of women, LGBTQ+ individuals, migrants, refugees, and minorities, who are the most disadvantaged segments of society (Gutiérrez Rodríguez et al., 2018). Beyond preventing these targeted groups from accessing and exercising their rights, anti-gender politics can incite overt acts of violence. Turkey exemplifies the rise of anti-genderism linked to top-down politics, illustrating a different trajectory compared to many Western countries. I contend that anti-gender politics in Turkey are predominantly top-down and are integrated into social engineering processes within a majoritarian-authoritarian-securitarian (MAS) political framework. At the same time, despite the support of micropower foci (e.g., fundamentalist religious communities, tariqats, feudal and tribal networks, town notables and fellow countrymen clubs in cities) and the anti-rights front for the anti-gender politics^1^, their grassroots reception remains limited (Yetiş & Özdüzen, 2024). Nonetheless, it is equally challenging to claim that substantial grassroots resistance exists against such politics; thus, the lack of such resistance allows these political climates to persist. Moreover, the global rise in anti-genderism, coupled with authoritarian tendencies, fosters similar political climates in different local contexts. Therefore, I assert that gendered violence should also be reconsidered within the contexts where anti-genderism has arisen and subsequently exacerbated it (Yetiş, 2025a).

In this article, I utilise the concept of *masculinist entrenchment*, previously developed to understand the ongoing top-down imposition of anti-genderism in Turkey (Yetiş & Özdüzen, 2024). The term acknowledges the deep-rooted and often institutionalised dominance of masculine norms and values, encompassing the structures, practices, and identities that reinforce and perpetuate gender inequality and gendered power dynamics in society and politics. Thus, rather than referring to efforts aimed at reclaiming and reinstating traditional masculine roles and values perceived to have been lost or diminished [as suggested by the concept of masculinist restoration (Kancı et al., 2023; Kandiyoti, 2021; Unal, 2021)], masculinist entrenchment becomes relevant where traditional masculine norms are deeply ingrained and already dominant in MAS politics. However, it is essential to note that the masculinist entrenchment described here is not exclusive to the gender regime in Turkey, which is also far from being static and unchangeable, harbouring complex paths toward both possibilities for and challenges against gender equality and justice, as I will continue to unfurl its intricacies. I argue that the more nuanced understanding of masculinist entrenchment underpins the top-down anti-gender politics that strategically capitalises on masculinist protection (Young, 2003), aligned with the discourses of victimhood and self-preservation of the so-called authentic national identity. This is achieved through the enactment of swashbuckling masculinity, which provides a sense of righteous aggression and violence to protect the family, state, and nation (Yetiş & Özdüzen, 2024), as part of *internalised domination*.

In these circumstances, the MAS political agenda in Turkey creates and sustains polarisation (Arat & Pamuk, 2019), which also “defies facile categorisation based on gender since both men and women may find themselves on the opposite side of this divide” (Kandiyoti, 2021, p. 215). Thus, as an unintended consequence of this polarisation, social justice activism focused on gender issues has become a common ground for critical scholars and a wider social movement to combat such political agendas, advocating for egalitarian, social justice-oriented, democratic transformations (Kancı et al., 2023; Özbay & Ipekci, 2024). A transformative approach can strengthen the feminist social justice framework by highlighting the visibility, power, and autonomy of women’s and LGBTQ+ movements. Feminist social justice, in relation to violence, means confronting both the visible and invisible forms of harm that sustain inequalities and injustices, hereby transforming them through inclusive, intersectional, and accountable practices. Accordingly, it is even more essential to consider men’s engagement in and interactions with these movements, especially since top-down anti-gender politics pose a threat to these initiatives and diminish their ability to connect with broader society through state-sanctioned punitive violence directed at them. However, the rise in social violence and ongoing gendered violence, both occurring with impunity, amplifies feelings of disgust and revulsion toward such violence (Bas et al., 2022; Yetiş, 2025b). Moreover, state-sanctioned punitive violence, supported by the MAS political regime in Turkey, reinforces these feelings and elicits a degree of socio-political criticism of social injustices (McManus, 2025; Yetiş & Kolluoğlu, 2022). Although this condemnation may pave the way for a more profound critique of social injustices, it is also likely to be ineffective at fostering meaningful engagement in social justice endeavours (Yetiş, 2025b; Yetiş & Kolluoğlu, 2022; Yetiş & Özdüzen, 2024). Thus, despite the apparent discontent towards the ongoing top-down anti-genderism and MAS political agenda in Turkey, which signifies potential for men’s engagement in social justice endeavours, it is crucial to recognise that this will not occur straightforwardly as men may find it challenging to adopt a feminist and gender-sensitive perspective due to their contentious social positionalities regarding power relations that involve dynamics of *internalised oppression and domination*, which I will elaborate on further in this article. In line with a feminist social justice-oriented approach drawing on the analytical framework of continuum thinking (Boyle, 2024), the article primarily aims to explore the challenging dynamics that obstruct men from collaborating towards a lasting societal transformation for social justice, gender equality, and the prevention of gendered violence. The analytical framework presented in this paper can foster the necessary sociological and political imagination to attain a comprehensive understanding of gendered violence, as well as alternative empowering visions with a robust political-ethical stance to resist top-down anti-gender politics couched within authoritarian populist political imagination (Yetiş, 2025c).

The literature pertaining to men’s relationship with anti-genderism predominantly focuses on analysing men’s role as mere supporters, either actively or passively, due to their affinity for anti-gender politics (e.g., Blais, 2021; Johanssen, 2021; Kaiser, 2022; Maricourt & Burrell, 2022; Roose & Cook, 2025; Sauer, 2020), often framed through narratives of, for instance, emasculation and victimhood attributed to feminism’s success, fears of reproductive threat posed by racialised others undermining majority men’s virility, the portrayal of gender as an ideological danger to the “natural” family and social order, anti-feminism as both anti-establishment and anti-colonial resistance, and the idea of men “reclaiming” power as a project of personal healing and societal salvation. However, there remains a gap for developing counter-narratives to mobilise men in support of feminist social justice and societal transformation toward gender equality and non-violence (MenEngage, 2025). I seek to partially close this gap in the existing literature and support the development of such counter-narratives by contributing to the academic discussion regarding both potentials and obstacles for men in resisting anti-genderism through the analysis of the intricate dynamics of internalised oppression and domination. I believe Turkey presents a distinctive context characterised by ongoing top-down anti-genderism, and this article examines such barriers and opportunities both within and beyond this context to address the globally escalating phenomenon of anti-genderism.

In pursuit of this aim, the paper primarily draws from my research on Turkey, including (1) men’s perceptions, thoughts and experiences of gendered violence (Yetiş, 2019), (2) the psychosocial approach to interviewing men on gendered violence (Yetiş, 2020), (3) male front-line workers’ challenges and opportunities in addressing men’s violence (Yetiş & Kolluoğlu, 2022), and (4) top-down anti-genderism and masculinist entrenchment in Turkey (Yetiş & Özdüzen, 2024).^2^ Through a re-analysis of these studies using various theoretical concepts with an interpretive approach (Heaton, 2004; Köhler et al., 2025), this article aims to provide a robust theoretical contribution and novel insights into what hinders men’s engagement in societal transformation towards feminist social justice within a context characterised by masculinist entrenchment (i.e., Turkey). Re-analysis is a methodological approach that can yield new theoretical insights from multiple studies that cannot easily be generated within the scope of a single study. Accordingly, this article presents an original analytical framework drawing on continuum thinking that links gendered violence and top-down anti-genderism with the intricate roles of internalised oppression and domination, in order to reveal what hinders men’s engagement in resisting such violence.

The arguments in this article are not based solely on the empirical data and concepts from these four studies, which have already been published elsewhere, but rather on an interpretive approach that draws on continuum thinking to a novel analysis of the intricate dynamic between internalised oppression and domination. Firstly, I present the concept of *continuum thinking* to comprehend the *conducive context* that enables a range of interlinked forms and multilayered repercussions of gendered violence, as well as the prevailing anti-gender politics within masculinist entrenchment. Following this *continuum thinking*, I discuss how the complex dynamic between *internalised oppression* and *domination* prevents men from developing alternative imaginations for a political-ethical stance that can facilitate social justice endeavours committed to non-violence.

## Continuum Thinking as an Analytical Framework for Gendered Violence

I argue that violence can be viewed as anything that obstructs the realisation of individuals’ and communities’ capabilities (Yetiş & Kolluoğlu, 2022). By this definition, we can broaden its meaning, making it far more comprehensive, interpretable, and politically charged. Following this logic, gender inequality can be seen as the main reason for impeding the capabilities of women, which also paves the way for different forms of gendered violence. However, as Walby et al. (2017) cautioned us, there is a risk of taking gender inequality as the most overarching form of gendered violence. This can lead to an overdetermination that tends to dilute the very definition of violence without differentiating between its various forms and the repercussions it can generate. On the other hand, we also need to shy away from the very siloed definitions of and piecemeal responses to gendered violence since they can be counterproductive to understanding and resisting such violence occurring and transposing through different ramifications and dimensions within the social structure (Yetiş, 2025a). Here, the concept of *continuum thinking* provides an analytical framework with the necessary sociological and political imaginations to comprehend and confront interconnected forms of violence without treating them as equivalent or analogous.

*Continuum thinking* in relation to gendered violence draws on Liz Kelly’s (1987) conceptualisation of the continuum of violence against women, signifying multiple factors at play behind such ongoing violence beyond individual, interpersonal and stand-alone incidents of violence. These factors mainly include the culture of everyday sexism, women’s poverty and economic dependence, gender pay and pension gaps, unequal participation and representation in political life, unequal access to public services and common goods, and sexist stereotyping in the media (Kelly, 1987). As an analytical framework, *continuum thinking* makes it possible to distinguish between different forms of violence while revealing links between them, considers violence as existing on a spectrum from subtle forms of harassment to severe physical violence and helps understand how seemingly minor acts of aggression can escalate into more severe forms of violence (Boyle, 2024). It also highlights the normalisation of certain behaviours, enabling a culture of violence as a *conducive context* (Kelly, 2016). Here, we can locate top-down anti-gender politics within masculinist entrenchment in Turkey as the *conducive context* that enables both gendered violence in varied forms and a culture of violence fostered by the MAS political agenda (Yetiş, 2025a). Turkey’s recent withdrawal from the Convention on Preventing and Combating Violence against Women and Domestic Violence (widely known as the Istanbul Convention) by President Erdoğan’s decree in 2021, along with the subsequent attempts to undermine Law No. 6284 on the Prevention of Violence against Women, are prominent examples of how gendered violence is increasingly met with impunity, while punitive violence is enforced against dissidents who advocate for the Convention and the Law that guarantees legal protection from gendered violence (Yetiş & Özdüzen, 2024). Furthermore, we can assert that globally rising anti-genderism, coupled with authoritarian tendencies, further exacerbates the conditions in local contexts. More recently, for example, President Trump’s anti-DEI (i.e., diversity, equality, inclusion) rhetoric and its widespread top-down adoption in the US (Boso, 2025) are likely to impact other local contexts as well, precipitating the attacks against women and LGBTQ+ movements, ethnic/racial minorities and immigrants and cutting their ties with broader society. Thus, contemporary politics in the global arena can also be seen as a broader conducive context in terms of continuum thinking, while recognising that both the contextual differences and the continuum between them are required to cultivate grassroots global political solidarity against these top-down authoritarian politics. In the face of these developments, both locally and globally, genuine engagement of men becomes even more vital for social activism against anti-gender politics. This is not just due to men being potential proponents for these politics, but also to enhance the link between the feminist social justice movement and society at large, thereby strengthening collective social action against these politics.

To facilitate this engagement by fostering social activism against anti-genderism*, continuum thinking* can provide a more comprehensive, intersectional, and inclusive understanding of gendered violence with different dimensions and forms, which goes beyond the binary thinking of men’s violence against women. Even though gendered violence is primarily conceptualised as violence against women, *continuum thinking* critically expands its definition, including violence against LGBTQ+ and gender non-conforming individuals, violence directed towards men and boys, violence between men, as well as the ongoing militaristic culture and practices both in times of conflict and during everyday life in so-called peaceful times (Boyle, 2024; Cockburn, 2010; Graaff, 2021). In this way, the definition of gendered violence includes not only acts of harm directed at people based on their gender or perceived gender - widely defined as gender-based violence - but also emphasises the gendered dimensions of all types of violence. It highlights how violence is shaped by and perpetuates gender norms and hierarchies, revealing the systemic and patterned nature of violence across various contexts. Following *continuum thinking*, we not only embrace a more comprehensive understanding of gendered violence but can also attain insight into how the different forms of social violence and injustices are imbued with gendered violence in a *conducive context* of anti-gender politics and how men are involved in these. Upon developing such insight, it becomes more possible to contemplate how men can engage in social action resisting them.

To resist gendered violence amid anti-gender politics*, continuum thinking* also paves the way for a feminist understanding of social justice, emphasising the importance of agency and its empowerment through the process of such resistance. By recognising the spectrum of violence, we can develop a more nuanced understanding of advocacy and interventions in pursuit of social justice that align with empowering individuals to challenge and resist violence at all levels. Once again, this underlines the importance of understanding that experiences of violence are influenced by intersectional and compounding factors such as gender, race, ethnicity, age, class, and sexuality, as well as being bodily or mentally abled/disabled (Crenshaw et al., 2024; Hill Collins, 2023; Hill Collins & Bilge, 2020). This helps develop more inclusive and effective interventions that address the diverse needs and potentials of individuals and communities. However, the empowerment perspective is not limited to the potential of utilising or attaining various resources to exercise their agency; it also involves cultivating alternative imaginations to develop a political-ethical stance (Pease, 2022) that can pave the way for social justice endeavours to commit to non-violence. In order to cultivate such imaginations, we need first to understand what is getting in the way. To address this, I put forward the analytical framework of *continuum thinking* to examine how the thorny dynamic between *internalised oppression* and *domination* hinders men from cultivating such imaginations. As noted in the introduction, dissatisfaction with the prevailing top-down anti-genderism and MAS political agenda in Turkey, which is rooted in masculinist entrenchment, holds the potential for men to engage in social justice endeavours by initiating socio-political critiques of ongoing injustices and violence that affect both them and others, albeit in different ways and at varying levels. However, it is also crucial to understand that this engagement will not happen in a straightforward manner. Thus, I hope this discussion presented here will facilitate the realisation of this potential by presenting transformative pathways.

## Power Relations, Intersectionality and Internalised Oppression in a Conducive Context

Although some scholars caution against conflating oppression with violence or carelessly mixing various types of physical and symbolic violence to prevent reductionism and conceptual confusion (Eriksson & Pringle, 2024; Walby, 2023), I believe it is still helpful to emphasise the connection between these concepts within the framework of *continuum thinking*. The connection assists in locating different forms of oppression in a *conducive context,* enabling different forms of violence, if not directly causing them. Symbolic violence (Bourdieu & Wacquant, 1992), for example, pertains to the internalisation of these oppressions, making the recognition, representation, and confrontation of different forms of violence increasingly difficult. Here, it is essential to consider internalisation as an indication of an intricate process rather than a fixed position in a given situation. For instance, when we regard men solely as agents of oppression, exploitation, and violence within a single gendered category, we risk promoting a reductionist account of masculinity that defines them merely as holders of power and status as oppressors. Thus, the very idea that men assert their masculinity by dominating women, either simply because they are men or only through the oppression they impose, rests on a tautological logic and consequently creates an ontological impasse that hinders the development of an alternative political-ethical stance for men to resist ongoing gendered violence. This viewpoint enforces a binary framework in which women are portrayed as victims and men as perpetrators, perpetuating symbolic violence. Such a perspective contradicts the *continuum thinking* by compromising a nuanced intersectional approach (Yetiş, 2025d), hindering our ability to envision the historical, social, cultural, and political transformations at play.

The various intersectional inequalities, historical and cultural differences, and individual experiences and personal characteristics that shape men’s lives and distinguish them from one another can be seen as both opportunities for and obstacles to social transformation in favour of gender equality and against gendered violence. Seizing opportunities and overcoming obstacles requires recognising differences and inequalities among men and assessing their potential for social transformation through an intersectional lens. Connell (2020) adeptly unfolds variations among men in their changing positions in the social hierarchy and existing power relations, highlighting complicit, marginalised, and subordinate masculinities and demonstrating how these different positions take shape in relation to hegemonic masculinity. However, while this provides critical studies on men and masculinities with the possibility of intersectional analysis, the main focus remains on hegemonic masculinity framing men’s domination over women and other marginalised/subordinate men as the modus operandi of patriarchal society. Strengthening the intersectional approach, we can also begin to understand how men have been oppressed and how they internalise that oppression, which can pave the way for engaging in transformative social justice endeavours to resist both their own and others’ oppression (Flood, 2019). Therefore, interrogating non-hegemonic experiences and expressions of different masculinities, rather than the glorified and idealised version of hegemonic masculinity, along with their contentious positions within a patriarchal society, can foster men’s participation in transformative social justice endeavours.

I apply Bourdieu’s concept of *symbolic violence* (Bourdieu & Wacquant, 1992), characterised by misrecognition and naturalisation, to examine the normalised nature of violence in power dynamics. Bourdieu points out that oppressed groups often internalise their conditions as normal due to misrecognising extant inequalities (Swartz, 2013). Misrecognition affects both the subordinated and dominant, though differently, involving complicity, internalisation, and embodiment. Symbolic violence manifests as *internalised oppression* among social agents, appearing as resignation and learned helplessness (often masked by unrecognised anger), which enables ongoing social and environmental harms, exploitation, discrimination, and exclusion (Yetiş & Bakırlıoğlu, 2023). According to this, it becomes possible, at least partially, to explain men’s condonation of violence and oppressive practices as unintended consequences of the *internalised oppression* they experience. Bishop (2015), for example, suggests that all oppressors have personally experienced oppression; otherwise, they would not allow themselves to become oppressors. If one were to follow this argument, it could be suggested that men might first need to address their own oppression and seek to demystify the symbolic violence they experience. By doing so, they might potentially develop greater empathy and sensitivity towards other forms of oppression, including those in which they are perpetrators or complicit. In terms of *continuum thinking*, an ongoing inquiry into *internalised oppression* is a commendable way to unfold different forms and dimensions of oppression that men are both exposed to and involved in. This is also evident in my studies (Yetiş, 2019, 2020; Yetiş & Kolluoğlu, 2022), which show that male participants can develop a sense of critical reflexivity by uncovering the oppression and violence they are exposed to and further reflecting on their own oppressive practices. This process potentially fosters aspirations for involvement in social justice campaigns and a feminist political solidarity, which is also characterised by a sense of openness and attentiveness to others’ oppression and suffering (Scholz, 2008).

The experiences of oppression, such as being a victim or witness, including domestic violence and abuse during childhood and youth, can initiate critical questioning of the harmful effects and impairments of such experiences, both in their lives and in others. Several frontline worker participants in my study (Yetiş & Kolluoğlu, 2022) specifically highlighted how such experiences significantly influenced their career choices in frontline services focused on protecting against domestic abuse and gender-based violence, aiming to be part of the solutions to problems they were already familiar with. Besides individual experiences, my studies with men (Yetiş, 2019, 2020; Yetiş & Kolluoğlu, 2022) also illustrate that living under oppressive and authoritarian socio-political conditions with the intensification of social injustices under the top-down MAS political agenda (socio-economic deprivation and political misrepresentation of marginalised communities of poor, minorities and immigrants), state brutality via police violence targeting politically dissident groups (especially women and LGBTQ+ groups), and prevalent arbitrariness of social violence with increasing impunity, all of which cause moral injuries, can instigate their critical thinking on such oppressive situations. As a result, this can also harbour a potential for their engagement in correcting such injustices to some extent.

Having acknowledged its potential advantages for feminist social justice endeavours, particularly in intersectional analysis of power relations and inclusivity for broader, robust social action, the stand-alone inquiries into internalised oppression of non-hegemonic masculinities can also be counterproductive, as they call forth some limitations in comprehending the more nuanced and intricate composition of power relations. First, it is much easier for those who occupy both oppressed and oppressor positions to concentrate on a struggle against the social conditions of their oppression but to ignore the other conditions enabling their privileged or oppressor status (Scholz, 2008). This is evident when the increasing gendered violence is merely regarded as epiphenomenal to broader social injustices and violence. Some of the men in my studies (Yetiş, 2019; Yetiş & Kolluoğlu, 2022), for example, primarily complain about deteriorating economic conditions and widening social inequalities, alongside rampant authoritarianism and arbitrary social violence that goes unpunished, while overlooking the continuum of gendered violence in society that extends back to the point they began to identify as problematic.

Second, it can turn into a race to innocence in which the groups holding a relative privilege can underestimate the extent of their privilege and oppression by over-prioritising their own oppressed or aggrieved situations in comparison with others (Pease, 2022). In the scenario of being a victim of oppression turning into a benchmark for innocence, the claims to be the victim can risk conveying apprehensions more of *internalised domination* than of oppression. In contemporary global society, for example, men who are primarily from dominant groups holding heteronormative and ethnoracial majority citizenship status are assumed to be in a sort of crisis with feelings of being aggrieved and resented based on a perception of losing their entitled higher status by their gender and ethnicity (Kimmel, 2019). This is mainly explained away in connection with a relative improvement in women’s positions and an increment in the visibility of LGBTQ+ people and ethnoracial minorities in social, economic and political domains. However, with the global ascendancy of neoliberal hegemony, these men (like many others) also find themselves in precarious conditions with a loss of opportunities for secure jobs, increasing underemployment and a relative decrease in their academic achievements, contributing to the symptoms of being “left behind” as the fear of being dislodged from their privileged positions in society (Kerrigan et al., 2025). These symptoms are mostly associated with the reasons why these men are inclined to buy into anti-gender rhetoric and anti-immigration sentiments spearheaded by authoritarian far-right populist politics (Nightingale et al., 2023). However, here, the rhetoric of crisis risks being incorporated into the race to innocence, operating not only as an excuse for their perpetration and complicity in oppressive practices of misogyny, homophobia and racial harassment but also deflecting attention from structural deadlocks of neoliberal capitalism, racism and sexism with their harmful repercussions. In relation to this, the concept of “masculinity in crisis” offers a makeshift explanation that overrepresents certain groups of men from the Global North (Hübinette, 2019) while failing to adequately address masculinities in other contexts with different gender regimes, particularly where we do not observe a notable improvement in the status of women and LGBTQ+ individuals, such as in the case of Turkey^3^ (Yetiş & Özdüzen, 2024; Zürn & Schäfer, 2023). Instead, socio-economic and psychosocial deprivation, living under oppressive and violent conditions, social exclusion, and discrimination are shaped by the relevant conducive context. Consequently, asserting that masculinity-in-crisis is a globally prevalent phenomenon becomes more challenging. Depending on the context, all of these obstacles generated by contemporary precarious conditions can be seen as reasons that undermine capabilities and diminish people’s life chances in many respects and to varying degrees, thereby exacerbating feelings of aggravation.

Having said that, these feelings in the face of various obstacles cannot be self-explanatory for the oppressors' ignorance of their oppressive practices, since even the resources to be mobilised (e.g., economic, social, cultural, and symbolic capital) to cope with these obstacles are not equally distributed among the oppressed (Pease, 2022). More importantly, the benefit of the doubt for innocence is not provided equally in a given socio-political context, and the most marginalised groups with the least resources are still the most deprived of it. For instance, in Turkey, some male groups who identify themselves as “victim fathers” and “men who are victims of alimony” aim to organise around a male victim identity (Sallan Gül, 2019), spurred both by a top-down anti-gender political agenda and by appealing to the globally prevalent discourse of male victimisation (Yetiş & Özdüzen, 2024). Their primary assertion is that women’s legal rights concerning custody and alimony undermine their own rights over their children and exacerbate their economic autonomy, thereby rendering them victims. Some perpetrators of domestic violence have also claimed that false allegations by women result in men being victimised by the very laws intended to protect women from men’s violence, such as the removal of the accused from their home as a precautionary measure (Yetiş & Kolluoğlu, 2022). During the campaign against the Istanbul Convention, the front against women and LGBTQ+ rights extensively promoted and circulated this argument to generate a moral panic around male victimisation (Unal, 2021). However, regardless of the limited repercussions of such moral panic in society, the political power has tapped into the argument as aligning with masculinist protection, where aggrieved men become violent towards their partners out of desperation and depression. In an ironic twist, this argument surrounding male victimisation was cunningly transformed into a concern for female vulnerability to justify the withdrawal from the Convention under the pretext of safeguarding women from men’s lethal violence (Yetiş & Kolluoğlu, 2022). Even though these claims remain marginal in society, they are instrumentalised by the ruling power to enforce anti-gender politics at the expense of women’s rights (Yetiş & Özdüzen, 2024). Therefore, these claims of being a victim are likely to resonate more with internalised domination than oppression, as discussed further in the next section, which begets severe moral injuries on feminist social justice endeavours, which can be harder to amend.

Thirdly, contrary to Bishop’s (2015) assumption presented earlier, it is not imperative that all of those who occupy oppressor positions are themselves oppressed. The assumption that oppressors must have personally experienced oppression harbours the naturalistic fallacy that humans are inherently moral and good if not social conditions urge them to do bad things. However, crimes of the powerful (Lynes et al., 2024), such as exploitation, corruption, and human rights violations sanctioned by state agents, which present the greater share of ongoing oppression and harm, do not require their perpetrators to have been oppressed, which is evident in top-down authoritarian politics. It is also apparent that not all individuals who have been oppressed inevitably find themselves in an oppressor position either, while they struggle to endure their oppressive conditions. Notwithstanding, the reverse logic that humans are essentially evil and only pursue their self-interests is equally problematic, not least because it begets the *fatalistic normalisation* (Yetiş & Bakırlıoğlu, 2023) of hierarchical and unjust social structure as unavoidably prevailing. Hence, this can further the *internalisation of oppression* by entrenching symbolic violence operationalised in a *conducive context,* enabling different forms of violence.

Accordingly, becoming an oppressor or not rather depends on the conditions and situations grounded in power relations within a *conducive context* that renders oppressive practices possible, as well as on how individuals develop political-ethical stances against these conditions and situations. Thus, capabilities need to be envisioned to cultivate such an ethical-political stance to transform the social structure by dismantling the conditions and situations that enable oppression and domination. To accomplish this, we also need to interrogate the dynamics of *internalised domination* with different faces enshrined in the structure of masculinity-in-defence.

## Internalised Domination, Masculinity-In-Defence and Afflictive Condemnation

In addition to grasping the dynamics of *internalised oppression*, the intersectionality approach within the framework of *continuum thinking* provides an analytical lens for understanding *internalised domination*, especially when the associated harms are denied, overlooked, or misrecognised by the relatively dominant and socially privileged groups. At the same time, perpetrators from these groups are not held accountable since they remain hidden, or their harms are justified in self-righteous, entitled manners (Tappan, 2006). The former mystifies the agents responsible for the harms, and the latter indicates the entitlement to harm others claimed by dominant groups through the endorsement of their superiority and the prioritisation of their interests as part of the natural social order or under the pretext of protecting themselves, their families, communities, and society. Internalised domination, in relation to symbolic violence, is often manifested through societal biases in norms, such as racialised and gendered social orders, regulations, laws, social policies, and institutionalisation (e.g., the education system), as a consequence of groups with greater social power imposing their norms on subordinate groups (Swartz, 2013). Accordingly, Bourdieu explicates symbolic violence not only through the social suffering of subordinated groups but also through its wider mechanisms of misrecognised compliance, legitimisation, and even the concealment of dominant groups’ interests through symbolic power, thereby reducing the possibility of resistance to such violence (Swartz, 2013).

There is no doubt that many men internalise a sense of dominance from their social power bestowed upon them by their gender position and perform various acts to demonstrate this. Many areas of life, from business to education, sports to health, are intertwined with material-discursive formations (Hearn, 2015) that portray strong, competitive masculinity. Apart from its material reality across different areas of life, this sense of domination mainly rests on a fantasy of masculine power and its performative enactment, sustained by the very ways of denying one’s own vulnerability and even one’s mortality as a human (Brown, 1998). However, the fantasy of power built on such masculinity is intrinsically untenable and handicapped by its very claim to such power, and the denial of such vulnerability requires compelling repression in itself. This situation reveals a paradox at the heart of the fantasy of masculine power and its performative enactment, which is also likely to have damaging consequences. Accordingly, many stress-induced illnesses associated with the repression experienced by men, the difficulty of expressing feelings, and their unwillingness and failure to seek help in solving problems (Lohan, 2007) should be addressed together with the oppression, control, and violence they exert, alongside the damage they inflict on themselves and their environments (Hearn et al., 2022). The denial of vulnerability accompanying the sense of domination can also lead men to take a defensive position in the face of any potential threat to the maintenance of such a fantasy of masculine power (Yetiş, 2020). It can manifest itself directly in violence and aggression for some men, but also as indifference, disregard, cynicism, and nihilism for many others. By the very reason of this defensive position wrapped around internalised domination, men can find it difficult to contemplate the place of violence in their lives and take a genuine stand against it. By examining the very vulnerabilities and paradoxes in the construction of masculinity (Whitehead, 2002) related to *internalised domination*, it becomes possible to develop a much deeper and more comprehensive analysis of gendered violence in relation to wider forms of social violence prevalent in society.

Righteous aggression rooted in internalised domination, for example, reflects a gendered performance of swashbuckling masculinity, shaped through the involvement of both political power and vigilantist citizens invested in the masculinist entrenchment of the MAS agenda in Turkey (Yetiş & Özdüzen, 2024). This masculinity employs mafia tactics, using violence for self-preservation under the pretext of being a victim for various reasons, fuelling moral indignation for nationalist claims against political dissidents, whom they appoint as enemies based on circumstance. Such mafia tactics operate to obscure the demarcation between the public and private spheres, as well as between lawful and unlawful violence (Scheper-Hughes & Bourgois, 2004). Additionally, paramilitary violence may be tacitly supported by the state and legitimised through the shift towards authoritarian governance in conflict zones, as evident in Turkey’s Kurdish conflict (Isik, 2021). Even extralegal social violence can be regarded by various societal actors as “legitimate” and implicitly or explicitly endorsed by state authorities and citizens against perceived enemies of the state or the in-group (Kadıoğlu Polat, 2021; Saglam, 2021). Violence that disproportionately affects certain parties and groups might not be recognised as such, especially by supporters of incumbent governments who are tempted to blame their rivals for provoking these incidents (Toros & Birch, 2021). Saglam (2021), for example, provides a detailed account of the masculine world among Turkish nationalists, where men embody and enact the state through their involvement in social violence, e.g. vigilante surveillance of outsiders, inventing conspiratorial narratives of foreign plots, and lynching political activists. His analysis demonstrates that vigilante violence within this nationalist context does not weaken state authority but rather sustains it, expanding the state’s control over society through men’s bodies, which “become the interfaces, means, and agents of state power” (Saglam, 2021, p. 222). In this way, privileges ascribed to state agents, such as the enactment of violence, surveillance, and punishment, are entrusted to ordinary citizens who uphold law and order in the name of the state and nation. Saglam’s research also illustrates how state-sponsored moral-judicial hegemony becomes embedded in daily social life, supported by masculine subjects, and plays a crucial role in their interactions with state agents. Lynching, referring to political violence targeting individuals or groups and excusing mob violence against those groups by deploying an extensive vocabulary on nationalism, is also a prominent political strategy of the Turkish far right, predominantly backed by state authorities (Bora, 2008; Daub, 2024). Here, the privilege to enact arbitrary violence to maintain political hegemony is a central pillar in the execution of the MAS agenda. As such, it becomes possible to extend the assessment of the recent increase in social violence alongside the encouraging impunity policies bestowed upon those in or allied with political power, as a reflection of internalised domination. Given the ongoing verbal abuse (Altinoluk & Koca, 2022) and lynching attempts against migrant and refugee groups in both online and physical spaces, we can interpret the perceived socio-cultural and “biological” superiority of Turks over refugees as a gendered phenomenon (Ozduzen et al., 2021) in relation to masculinity-in-defence. In addition to asserting dominance over other groups, recourse to violence in any contentious situation faced in everyday life becomes feasible through the act of righteous aggression. This is also accompanied by a general rise in incidents of violence involving firearms and knives (Umut Vakfı, 2024), as well as acts of violence against health workers, ranging from verbal to physical and sexual abuse (Demirci & Uǧurluoǧlu, 2020). Although these incidents of violence are not necessarily directly supported by political power, the cultural climate of violence fostered by top-down MAS politics has encouraged such occurrences.

Drawing on righteous aggression with the performance of swashbuckling masculinity, enactment of violence (either by state agents or ordinary citizens) couched in anti-gender politics cannot be explained away as merely a reaction to the advancements related to gender equality, women’s empowerment, LGBTQ+ visibility, and the possible erosion of traditional gender roles and expectations. Other than being reactive, violence is predominantly regarded as a right bestowed upon men and an obligation they must fulfil when deemed necessary, including the defence of their home, family, honour, homeland, and nation (Yetiş & Kolluoğlu, 2022). A form of masculinity that utilises violence is cultivated for the defensive and protective imperatives of securitarian politics that legitimise violence as a means or resource (Brown, 2010). The roles ascribed to this masculinity are characterised by maintaining and assuring order, preventing chaos, and avoiding perceived threats. Immanent to the hegemonic formulation of masculinity (Connell & Messerschmidt, 2005), these roles are consistently reinforced through an ongoing relationship between militarism and masculinity that elucidates the broader social acceptance of men’s “righteous enactment of violence” (Sjoberg, 2014). Accordingly, it is widely anticipated that men are prepared to resort to violence to defend their (material, symbolic and imaginary) possessions, and particularly to embody masculinity as a revered value and a symbol of power (Bourdieu, 2002). In this scenario, masculinity-in-defence refers to such a paradox foundational in the psychosocial construction of masculinity, inherent to internalised domination. However, because of the paradox, masculinity-in-defence can also be transformed into a gendered political performativity in which men strategically claim “victim status” when they attempt to attain what they believe they are entitled to (Yetiş, 2020). It is globally evident that such a masculine subjectivity-in-defence is stimulated by right-wing, conservative, and nationalist politics, along with authoritarian populist governments that seek refuge in securitarian politics. These entities aim to provoke this masculinity-in-defence in order to rally support for their agendas (Greig, 2019). When it comes to anti-gender politics in Turkey, this showcases how political decisions and actions regarding gender are underpinned by a punishment mechanism established and supported by political power through masculinist entrenchment. An imagined conservative family concept is mobilised here, depicted as threatened by LGBTQ+ people and women’s rights advocates. Consequently, political power can accuse these groups of disrupting society’s fundamental values and deploy extra-legal instruments to demonise LGBTQ+ activists and certain feminist groups by separating their political struggle from each other and other oppositional groups (Ozbay, 2021; Zengin, 2024). Therefore, to resist anti-gender politics and promote feminist social justice, it becomes essential to dismantle such masculinity-in-defence, which also warrants further analysis beyond its performative swashbuckling form.

When we apply *continuum thinking* to understand this defensive attitude with all its complexities, we can see that such an attitude is not confined to those who embrace swashbuckling masculinity through overt acts of violence; it also hampers many other men from having the courage to reflect on their own masculinity, voice their concerns, and question their actions, which implicitly condone violence to persist in their lives and those of others. Violence, therefore, remains a subject that men mostly shy away from discussing, or when they do speak about it, they instead adopt an abstract, impersonal yet normative stance that involves *afflictive condemnation* (Yetiş & Bakırlıoğlu, 2023) by externalising it as other men’s problem or as an overarching systemic issue, and as part of other problems generated by ruling power and their authoritarian politics, which encourage violence through ongoing impunity.

As evident in my studies (Yetiş, 2019; Yetiş & Kolluoğlu, 2022), as in many others (e.g., Berggren & Gottzén, 2022; Hearn, 2010), men rarely demonstrate outright support for violence. On the contrary, they principally condemn violence as an uncivilised practice while provisionally condoning it in situations when they feel themselves or their families, communities and society are perceived as under threat. At other times, violent action is regarded as shameful and requires to be punished harshly. Increasing social violence, in parallel with gendered violence, faced with impunity in Turkey, further augments the feelings of disgust and repulsion against such violence. In addition, state-sanctioned punitive violence against political dissidents under the tutelage of incumbent political power strengthens these feelings with a pinch of political criticism of social injustices (Yetiş, 2019; Yetiş & Kolluoğlu, 2022). While this condemnation can provide a path towards further criticism of social injustices, it is also inclined to be inconsequential for their sufficient engagement in social justice endeavours, especially when it serves to wash their hands of responsibility for ongoing injustices and violence. Thus, I define this condemnation as afflictive, mainly because of its overall inefficacy in social action against social injustices and gendered violence; however, I also acknowledge that individual intentions and motivations may vary and that it is nearly impossible to understand their true nature.

*Afflictive condemnation* here also involves projecting violence as “the other man’s problem”, who are considered “losers”, emotionally unstable, socio-economically and socio-culturally powerless men (Yetiş, 2019). These include uneducated, addicted, unemployed (mostly young) men whom my participants perceived as incapable of achieving a provider role or self-sufficient masculine ideals. The condemnation of violence remains perfunctory as long as it keeps endorsing the equation of a “virtuous” masculinity with the status of a power holder. Hence, such condemnation aligns with the assumption that the lack of social power inevitably drives men into desperation, leading them to enact violence as grasping at straws (e.g., Hall, 2002). This condemnation enables these men to save face by not appearing as powerless as other men who are presumably prone to violence, if not ensuring a pathway to becoming a fully-fledged power holder or a self-sufficient, respectable man. Nonetheless, this also reaches beyond its commonsense reception and finds a place in scholarly explanations of men’s violence (e.g. Messerschmidt, 2014), which overwhelmingly stresses the connection between relatively powerless men who are deprived of other resources to attain respectful status in society and violence as the mere resource left for these men to perform their masculinity. Yet such explanations are still enshrined in a tautological logic since they cannot help but explain such violence as an intrinsically gendered expression of men who are or feel powerless. In this way, the explanation unwittingly reproduces the masculine understanding of power with its paradoxical nature, that men ideally must achieve social power with respectable status; otherwise, it would indicate a crisis (of masculinity) being experienced either individually or collectively and would pave the way for the enactment of violence as an (almost unavoidable) expression of such a crisis. Once again, this conundrum can be addressed through *continuum thinking*, not least because men’s violence can be linked to both positions of having power and not having it, as these positions are constituted relative to each other and are not mutually exclusive. Thus, we mostly find these positions intermingled in everyday life, and their meanings are open to interpretation depending on the situations that enable violence. Moreover, there are other forms of violence at play that are not always vocally condemned or problematised in the same manner as the violence of those supposedly without power (Rawlings, 2021). These forms are often hidden, either condoned as acceptable gendered norms and expressions of protecting family, community, and nation, or, due to a lack of individual accountability, resulting in collective complicity and the diffusion of responsibility (as observed in state violence and lynching discussed above), which makes their immediate effects hard to see over time. These forms are highly resonant with masculinity-in-defence and also reflect indirect yet intertwined forms of violence entrenched in the social structure, including militaristic nationalism and anti-immigrant sentiments, alongside persistent gendered violence in various forms, which nevertheless reinforce one another along a continuum.

Accordingly, such condemnation also involves another form of projection through a grouping mechanism overwhelmingly targeting men who are located on the margins of society, as they come from backgrounds different from the dominant identity groups, such as immigrants and minorities (Scheibelhofer, 2017). In my studies (Yetiş, 2019; Yetiş & Kolluoğlu, 2022), for example, Kurdish men as a politically and socially marginalised minority group and Arab men as immigrants in Turkey are sometimes depicted as violent masculinities and posing a threat to women and girls, families and the whole social order. On the basis of orientalist imaginations, their cultures are condemned as being the pillar of traditional patriarchy that allows for the persecution of women, and those men are regarded as the leading actors of parochial sexist practices and attitudes that belong to their patriarchal culture. Such arguments are followed by statements that those men would need to be tamed; if not, they cannot be integrated into Turkish society and hence, should be punished harshly and eliminated (Yetiş, 2019; Yetiş & Kolluoğlu, 2022). The condemnation here resembles many Western countries in how non-Western, non-white men and their cultural backgrounds can be criminalised under the guise of protecting the nation, society, as well as women, while simultaneously neglecting to defend women’s rights in other spheres of social life (Farris, 2017). Ironically, the image of Turkish men is also confined to this usual-suspect profile within these Western nations (e.g., Aral & Juang, 2025; Kakavand & Trilling, 2022; Schaeffer, 2013; Verkuyten et al., 1995). However, this does not suggest that the portrayal of dangerous men is constructed arbitrarily; rather, it illustrates how this condemnation takes contemporary forms that reflect the socio-political conditions in a *conducive context* shaped by anti-immigration politics alongside ongoing racism and xenophobic nationalism within the logic of masculinist protection.

This condemnation becomes even more afflictive when we observe its function as absolving men coming from dominant and established insider groups by projecting the very home-grown problem of ongoing gendered violence onto “outsiders” as (imagined) external and intruder actors. The grouping mechanism based on the differentiation between the established and the outsider capitalises on increasing sensitivities in the face of the exacerbated issue of violence against women to reinforce moral panics around imagined “perilous masculinities” and triggers masculinist protection, lining up with ever-increasing anti-immigrant sentiments and patriotic feelings, which is conveyed by the entrenched position of masculinity-in-defence. Regardless of their political stance on either being proponents or opponents of the incumbent political power and its “violence regime” (Hearn et al., 2022), the activation of masculinity-in-defence goes beyond polarisation initiated by authoritarian populism. The nature of masculinity-in-defence consistently deflects attention from the path-dependent problems ingrained in the *conducive context* that enables such violence, and instead externalises the problems to somewhere else or some other groups in the form of afflictive condemnation. Therefore, the condemnation activated by masculinity-in-defence not only brings together conflicting political stances in the same form in a very ironic way but also tactlessly undermines the advocacy endeavours of women’s movements against ongoing gendered violence. Even if masculinity-in-defence does not always manifest itself directly in violent and aggressive forms, the afflictive condemnation as the other face of it overwhelmingly diminishes the potential for socio-political criticism against the rampant social violence, as it hinders any sense of openness and attentiveness to others’ oppression and suffering. Thus, the development of counter-narratives resisting anti-genderism requires also reckoning with afflictive condemnation in relation to masculinity-in-defence.

Nevertheless, *internalised domination* enshrined in masculinity-in-defence is not a universal mechanism that operates uniformly in all men. This would have been no different from essentialist and reductionist approaches that attempt to explain the entirety of men’s experiences through an all-encompassing conceptualisation of masculinity, much like those I criticise. While it is impossible and unnecessary to establish a complete definition of masculinity that encompasses all the varied, incoherent, and mutable experiences of men, within the framework of continuum thinking, it is still sensible to argue that the masculine fantasy of power, rooted in internalised domination, continues to significantly influence these diverse and sometimes conflicting experiences. Similarly, as the meaning and function of this mechanism for men are called into question, so too are the social relations founded on a comparable fantasy of masculine power that perpetually reproduces it, along with the significance of the authority and hierarchy inherent in these relations. Therefore, it is vital to demonstrate how this mechanism corresponds with Turkey’s anti-gender politics as well as other contexts in the contemporary world.

## Conclusion

The aim of this study has been to explore how the intertwined dynamics of internalised oppression and internalised domination shape men’s positionalities in relation to gendered violence within contexts of top-down anti-gender politics, with Turkey serving as a critical case. By applying the analytical lens of continuum thinking, the study has sought to reveal how violence operates across a spectrum from everyday sexism and symbolic violence to overt punitive aggression and how these forms are exacerbated by authoritarian, majoritarian, and securitarian political agendas.

This article has argued that Turkey’s top-down anti-gender politics, embedded in masculinist entrenchment, sustains and legitimises gendered violence. By employing continuum thinking, the analysis has demonstrated that violence must be understood not only in its overt forms but also in its symbolic, structural, and everyday manifestations. Within this conducive context, the ways for men’s engagement in feminist social justice are convoluted by the dual dynamics of internalised oppression and internalised domination. The framework developed here highlights that men’s positionalities are neither static nor uniform: they are shaped by intersectional inequalities, historical trajectories, and socio-political conditions that render them both potential allies and obstacles in struggles against anti-genderism. While reckoning with internalised oppression can potentially open pathways to critical reflexivity on violence, it can also risk devolving into a race to innocence that absolves responsibility. Internalised domination is enshrined in masculinity-in-defence, on the one side, operating to legitimise righteous aggression and normalising violence through a performance of swashbuckling masculinity, and on the other side, embracing an afflictive condemnation through denouncement of violence in abstract terms while simultaneously attributing its enactment and complicity to “other” men, often those perceived as socially marginal, unstable, or culturally different, thereby externalising responsibility and absolving themselves of accountability. Together, these processes reveal why discontent with social and gendered violence does not automatically translate into transformative engagement toward feminist justice, resisting top-down anti-gender politics.

While the literature pertaining to men’s affinity with anti-gender politics predominantly focuses on analysing men’s role as mere supporters, either actively or passively, there remains a need for developing counter-narratives to mobilise men in support of feminist social justice and societal transformation against such politics. In this article, I seek to partially close this gap in the existing literature and support the development of such counter-narratives. Accordingly, the contribution of this study lies in providing a nuanced analytical framework of continuum thinking that links gendered violence, anti-gender politics, and the contentious roles of various forms of masculinities within them. By applying this analytical framework across Turkey’s political landscape, the study highlights that anti-gender politics are not just populist instruments rooted in backlash politics, but also deeply embedded in coercive strategies of authoritarian rule. This embeddedness involves state-sanctioned punitive violence posing a constant existential threat to women’s and LGBTQ+ movements and diminishing their ability to connect with broader society, making them a soft spot exploited by authoritarian politics. It emphasises the importance of men’s involvement in and engagement with these movements. Developing counter-narratives in this pursuit must reckon with both internalised oppression and domination, dismantling masculinity-in-defence while cultivating alternative political-ethical imaginations committed to non-violence. Such efforts require inclusive, intersectional strategies that foreground the agency of women, LGBTQ+ people, and other marginalised groups, while also mobilising men beyond the paradoxes of masculine understanding of power.

## Data Availability

Due to the sensitive nature of the research and ethical concerns, supporting data is not available*.

## Notes

1. This article does not specifically focus on these anti-gender actors; however, there are various studies that zoom in on various grassroots-level anti-gender actors, such as Alnıaçık and Altan-Olcay (2025), Unal (2024), and Eslen-Ziya (2022), among others. We previously presented a more comprehensive and nuanced analysis of these groups (as micropower foci and anti-rights front) in relation to top-down anti-gender politics in Yetiş and Özdüzen (2024).

2. Yetiş (2019) is a four-year fieldwork in Turkey, based on in-depth interviews with 34 men, to explore men’s experiences and perceptions of violence. Yetiş (2020) is a conceptual review on the methodological implications of the psychosocial approach in interviewing men on gendered violence. Yetiş and Kolluoğlu (2022) is a one-year project, involving three focus group studies with male social workers in Turkey, to understand their opportunities and challenges in addressing men’s violence. Yetiş and Özdüzen (2024) is a critical feminist case study analysing anti-gender politics in Turkey within the broader context of global neoliberal and authoritarian transformations.

3. The latest data from Integrated Values Surveys (2024) between 1993 and 2022 shows that the perception of homosexuality has not immensely progressed in Turkey. The percentage of people finding homosexuality not justifiable [cumulative responses of 1 to 4 in a scale from 1 (never justifiable) to 10 (always justifiable)] only dropped appx. 5% in the past three decades (from 89.9% to 84.1%), and remains the highest in Europe. By comparison, even the countries perceived to have stronger anti-gender movements demonstrate larger improvements, such as Poland (from 85.4% to 56.1%) and Hungary (from 75.8% to 58%). It is evident that anti-LBGTQ + politics is not only an instrument for polarisation (like other authoritarian populist governments) but rather a soft spot to hinder potential alliances against top-down anti-gender politics in Turkey. This is the very reason why political power is tapping into that spot by spearheading a moral panic around the rights and visibility of LGBTQ+ people and organisations. However, this situation, putting LGBTQ+ people and organisations at risk of increasing punitive state violence, cannot be explained away through backlash against LGBTQ+ visibility, since there hasn’t been any significant improvement in LGBTQ+ rights either despite growing grassroots activism. Instead, this explicates the ongoing hesitance and reluctance of wider civil society activists and oppositional political parties when it comes to standing with LGBTQ+ people and advocating for their rights.
